# Binary and Ternary 3D Nanobundles Metal Oxides Functionalized Carbon Xerogels as Electrocatalysts toward Oxygen Reduction Reaction

**DOI:** 10.3390/ma13163531

**Published:** 2020-08-10

**Authors:** Abdalla Abdelwahab, Francisco Carrasco-Marín, Agustín F. Pérez-Cadenas

**Affiliations:** 1 Materials Science and Nanotechnology Department, Faculty of Postgraduate Studies for Advanced Sciences, Beni-Suef University, Beni-Suef 62511, Egypt; 2 Carbon Materials Research Group, Department of Inorganic Chemistry, Faculty of Sciences, University of Granada, Campus Fuentenueva s/n, 18071 Granada, Spain; fmarin@ugr.es; 3 Unit of Excellence in Chemistry Applied to Biomedicine and the Environment, University of Granada, 18071 Granada, Spain

**Keywords:** metal oxides, carbon xerogel, equivalent series resistance, oxygen reduction reaction

## Abstract

A series of carbon xerogels doped with cobalt, nickel, and iron have been prepared through the sol–gel method. The doped carbon xerogels were further functionalized with binary and ternary transition metal oxides containing Co, Ni, and Zn oxides by the hydrothermal method. A development in the mesopore volume is achieved for functionalized carbon xerogel doped with iron. However, in the functionalization of carbon xerogel with ternary metal oxides, a reduction in pore diameter and mesopore volume is found. In addition, all functionalized metal oxides/carbon are in the form of 3D nanobundles with different lengths and widths. The prepared samples have been tested as electrocatalysts for oxygen reduction reaction (ORR) in basic medium. All composites showed excellent oxygen reduction reaction activity; the low equivalent series resistance of the Zn–Ni–Co/Co–CX composite was especially remarkable, indicating high electronic conductivity. It has been established that the role of Zn in this type of metal oxides nanobundles-based ORR catalyst is not only positive, but its effect could be enhanced by the presence of Ni.

## 1. Introduction

The negative effects that fossil combustion has had upon the environment has created a demand to develop and implement alternative clean energy sources [1,2]. Fuel cells can dramatically decrease the greenhouse gas emissions because they are considered as a zero carbon emissions device. The proton exchange membrane (PEM) fuel cell is the most widely used fuel cell in potential applications such as vehicles and power units [3,4]. Unfortunately, the high cost per unit energy delivered reduces the use of PEM fuel cells at a large scale and introducing to market. This problem can be solved either by reducing the used fuel cost, typically H_2_, or searching for an electrocatalyst that enhances the overall efficiency and energy generation.

The fuel cell half reactions are an anodic hydrogen oxidation reaction (HOR) and cathodic oxygen reduction reaction (ORR). The overpotential for HOR is considered as negligible, while for ORR, it requires high energy to initiate the reaction due to large kinetic inhibition. Thus, the oxygen reduction reaction (ORR) is considered as the rate-determining step, and reducing its overpotential will enhance the fuel cell efficiency. It was found that the ORR can be carried out through a four-electron transfer pathway or a two-electron transfer pathway (see below). The four-electron transfer pathway is the favored one, because it forms the hydroxyl species rather than peroxide [5,6], which causes membrane degradation and electrode fouling. The ORR reactions in both acidic and basic medium can be summarized as follows [3,7].

Acidic media

O_2_ + 4H^+^ + 4 e^−^→2H_2_O direct (4 electron pathway) (1)

O_2_ + 2H^+^ + 2e^−^→H_2_O_2_ indirect (2 electron pathway) (2)

H_2_O_2_ + 2H^+^ + 2e^−^→2H_2_O (3)

Alkaline media

O_2_ + 2H_2_O + 4e^−^→4OH^−^ direct (4 electron pathway) (4)

O_2_ + H_2_O + 2e^−^→HO_2_^−^ + OH^−^ indirect (2 electron pathway) (5)

HO_2_^−^ + H_2_O + 2e^−^→3OH^−^ (6)

The peroxide formation can be avoided by using an effective electrocatalyst that forces the reaction toward the four-electron pathway. Recently, many advanced composites were tested as electrocatalysts for the reduction reaction in energy conversion technologies [8]. These advanced electrocatalysts are classified as platinum group metal (PGM), non-PGM catalysts, carbon-based catalysts, and single-atom-based catalysts. An example for PGM catalysts is Pt–Pd alloy or a core–shell structure [9], which show enhanced activity and durability for ORR. Platinum (Pt) and its alloys are considered as the best electocatalysts for ORR, which carries out the reaction through the 4-electron pathway with the formation of only water as the product [10,11,12]. However, the high cost and scarcity of platinum reduces its global use on a large scale on PEM. Recently, transition metal oxides-based electrocatalysts, whether mono-metal, binary-metal, or ternary-metal oxides, have become promising candidates for Pt-based materials in ORR application due to their active sites, high electronic conductivity, low cost, and environmental benignity [13,14]. However, the activity of transition metal oxides toward ORR is still unsatisfactory due to some reasons such as their lower electronic conductivities compared with Pt ones [15]. Improving the conductivity of transition metal oxides can be achieved through doping with an electron donor or by their encapsulation in a carbon matrix [16,17]. Jie Wang et al. synthesized an electrocatalyst of hollow-structured carbon-supported nickel cobaltite nanoparticles for oxygen reduction and evolution reactions [18]. In this work, vulcan XC-72 was used as the carbon source, and they found that the hollow structure of nickel cobaltite increases the number of active sites, which in turn increases the contact between the electrolyte and the catalyst, improving the ORR activity.

Recently, carbon gel [19,20,21], which is a novel carbon nanomaterial, has found applications in wide research areas due to its tunable surface area and porosity depending on the synthesis conditions [22,23,24]. Carbon gels in its both forms, aerogel and xerogel, were used in different fields such as catalysis [25], energy storage [26], and catalyst support in PEM fuel cells [27]. Different methods can be used for the functionalization of carbon xerogels with transition metal oxides. Haye et al. [28] prepared a ZnO/carbon xerogel composite as a photocatalyst by low-pressure plasma treatment and tested it for the Rhodamine B degradation. In addition, El-Deeb et al. [29] prepared a nickel cobaltite-decorated carbon xerogel composite (NiCo_2_O_4_/CX) for methanol electro-oxidation. The data show a good catalytic current density of 98 mA.cm^−2^ at 0.29 V with a stability that reaches 90.6% after 100 repetitive cycles.

Although numerous publications study the role of binary metal oxides on ORR activity, only a few reports study the effect of ternary transition metal oxides for ORR application [30]; especially, the role of Zn in this type of catalytic phase is still unknown. Herein, we report the synthesis of novel electrocatalysts: binary and ternary transition metal oxides-functionalized carbon xerogels.

In this work, a series of carbon xerogels doped with transition metals (M–CX i.e., M = Co, Ni, and Fe) were prepared as a cathode support of ternary 3D nanobundles based on Co, Ni, and Zn, to investigate the comparative electrocatalytic behavior of both transition metal-doped carbon supports and Zn-containing metal oxides on the reduction reaction of oxygen. The carbon xerogels doped with transition metals were prepared through a sol–gel method from resorcinol and formaldehyde monomers. After that, the doped carbon xerogels were further functionalized with binary and ternary transition metal oxides, forming nickel cobaltite (NiCo_2_O_4_), zinc cobaltite (ZnCo_2_O_4_), and zinc nickel cobalt oxide (Zn–Ni–Co oxide) phases, using a hydrothermal method. Finally, the developed composites materials were tested as electrocatalysts to oxygen reduction reaction in basic medium.

## 2. Materials and Methods

### 2.1. Materials

All used chemicals were purchased from Sigma Aldrich (Merck, Madrid, Spain), they are of analytical grade and used without further purification. These chemicals include resorcinol, formaldehyde (37 wt % in H_2_O), cobalt acetate, nickel acetate, iron acetate, urea, ethylene glycol, N, *N*-dimethylformamide, nafion solution (5 wt %), ethanol, isopropanol, and potassium hydroxide.

### 2.2. Preparation of Transition Metal Doped Carbon Xerogels (M-CX)

Metal-doped carbon xerogels were prepared from resorcinol and formaldehyde monomers through the sol–gel method published elsewhere [31]. Briefly, resorcinol (R) and formaldehyde (F) were mixed together with a molar ratio of 1:2 in the presence of water (W) as a solvent and metal acetate salt as the dopant (C). The molar ratio between R/W is 1:17 and the amount of metal dopant (M = Co, Ni and Fe) was calculated to be 1 wt % of the carbon matrix. After gelation, the obtained organic gels were subjected to the microwave drying process followed by the carbonization process in N_2_ atmosphere at 900 °C for two hours with a heating and cooling rate of 1 °C/min. The N_2_ atmosphere was kept on during the whole process.

### 2.3. Preparation of Binary (XY_2_O_4_/M-CX) and Ternary (X-Y-Z Oxide/M-CX) Transition Metal Oxides Functionalized Carbon Xerogel Composites

In this preparation, 80 mg of the prepared M-CX was dispersed in 40 mL of *N*,*N*-dimethylformamide (DMF) [32]. For NiCo_2_O_4_/M-CX, 0.5 mmol of Ni(Ac)_2_·6H_2_O, 1 mmol of Co(Ac)_2_·4H_2_O, and 6 mmol of urea were dissolved in 30 mL of H_2_O and ethylene glycol solution with a volumetric ratio of 2:1. The two solutions were mixed together and sonicated for 15 min; then, they were transferred into 100 mL Teflon lined stainless steel autoclave and the temperature was held at 180 °C for 12 h. After cooling down to room temperature, the powder was separated with centrifugation and then washed several times with water and ethanol, followed by drying at 60 °C for 12 h. Finally, the sample was calcined in air at 360 °C for 3 h. The same procedure was followed for the preparation of ZnCo_2_O_4_/Co-CX except for exchanging the nickel precursor for the zinc one. In addition, in the preparation of the ternary transition metal oxide/carbon xerogel (Zn-Ni-Co oxide/Co-CX), 0.5 mmol of zinc acetate, 0.5 mmol of nickel acetate, 1 mmol of cobalt acetate, and 6 mmol of urea were mixed together, and the same preparation steps were followed.

### 2.4. Characterization of the Prepared Materials

The surface areas and porosity of the prepared samples were characterized by N_2_-gas adsorption at −196 °C (Quadrasorb, Boynton Beach, FL, USA). The samples’ morphology was evaluated with scanning electron microscopy (SEM), Zeiss SUPRA40VP, (Carl Zeiss AG, Oberkochen, Germany). The samples’ particle size was determined by high-resolution transmission electron microscopy (HRTEM), FEI Titan G2 60–300 microscope (FEI, Eindhoven, The Netherlands). Samples’ crystallinity was characterized by X-ray diffraction (XRD) BRUKER D8 ADVANCE diffractometer (BRUKER, Rivas-Vaciamadrid, Spain). X-ray photoelectron spectroscopy (XPS) measurements were carried out with a Kratos Axis Ultra-DLD X-ray photoelectron spectrometer (Kratos Analytical Ltd., Kyoto, Japan).

### 2.5. Electrode Preparation

The working electrode was prepared by dispersing 5 mg of the prepared materials in 400 µL isopropanol and 30 µL nafion solution (5 wt %); then, it was sonicated for 10 min. Then, 10 µL of the prepared ink were deposited onto a rotating disk electrode (RDE) surface with a 3 mm glassy carbon tip and dried under an infrared lamp.

### 2.6. Electrochemical Measurements

All electrochemical measurements were performed using a biologic VMP3 (BioLogic, Seyssinet-Pariset, France) multichannel potentiostat with a standard three-electrode electrochemical cell in which an Ag/AgCl electrode was used as a reference, while Pt-wire was used as the counter electrode. Then, 0.1 M KOH was chosen to be the used electrolyte. Cyclic voltammetry (CV) was carried out in N_2_ and O_2_ saturated electrolyte in a potential window ranging from −0.8 to 0.4 V with two scan rates of 50 mV∙s^−1^ and 5 mV∙s^−1^. Meanwhile, linear sweep voltammograms (LSVs) were obtained in O_2_-saturated electrolyte with different rotation speeds (from 500 to 4000 rpm) at 5 mV∙s^−1^. Data obtained from LSV were fitted to a Koutecky–Levich model in order to determine the number of transferred electrons and the electrocatalytic performance of each sample as follows [33,34].

(7) $$\frac{1}{j}=\frac{1}{j_{k}}+\frac{1}{B\omega^{0.5}}$$

(8) $$B=0.2nF{\left(D_{O_{2}}\right)}^{2/3}\;v^{-1/6}C_{O_{2}}$$

where j is the current density; $j_{k}$ is the kinetic current density; ω is the rotation speed; *n* is the number of transferred electrons per O_2_ molecule; *F* is the Faraday constant; $D_{O_{2}}$ is the oxygen diffusion coefficient (1.9 × 10^−5^ cm^2^∙s^−1^); v is the viscosity (0.01 cm^2^∙s^−1^); and $C_{O_{2}}$ is the oxygen concentration (1.2 × 10^−6^ mol∙cm^−3^).

Electrochemical impedance spectroscopy (EIS) was performed in a two-electrode system in order to evaluate the electrode resistance and the equivalent series resistance. The applied frequency was ranging from 100 KHz to 1 mHz.

## 3. Results and Discussion

### 3.1. Physicochemical Properties

The porous texture of the samples was characterized with N_2_ gas adsorption. Before analysis, the samples were degassed at 100 °C for 12 h. Brunauer-Emmett-Teller, BET equation was applied to the obtained isotherms to calculate their corresponding surface areas [35]. The data obtained from N_2_ gas adsorption are compiled in Table 1. All the electrocatalysts show low S_BET_ surface areas, which range between 44 and 74 m^2^·g^−1^. Among the samples without Zn, the effect of Fe, Co, and Ni as metallic doping of the carbon support is directly proportional to the atomic radius of the metal, in which the NiCo_2_O_4_/Fe-CX sample is the one with the largest surfaces and pore volumes. However, it is very interesting that the Zn-containing composites have the lowest values of surface areas and pore volume, especially the Zn-Ni-Co/Co-CX sample.

**Figure 1:**
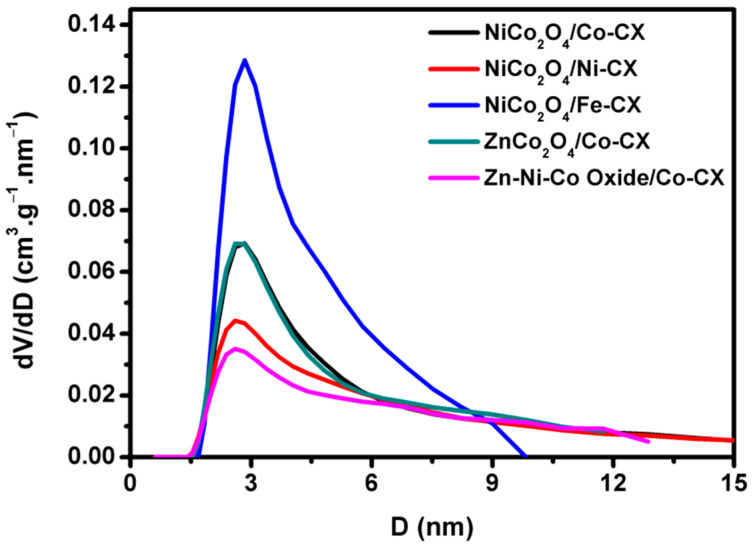
Pore size distribution from QSDFT analysis for the adsorption part of N_2_ isotherms.

Pore size distributions were determined by applying quenched solid density functional theory (QSDFT) to the N_2_ adsorption data (Figure 1) [36]. In this figure, we can observe that the main pore size of these composites is situated in the limit between the micropore and mesopore values, in which Zn–Ni–Co/Co–CX was the composite with a pore size distribution more shifted to the microporous range and the QSDFT surface areas (S_DFT_) were ranging from 79 to 201 m^2^.g^−1^, as shown in Table 1.

**Figure 2:**
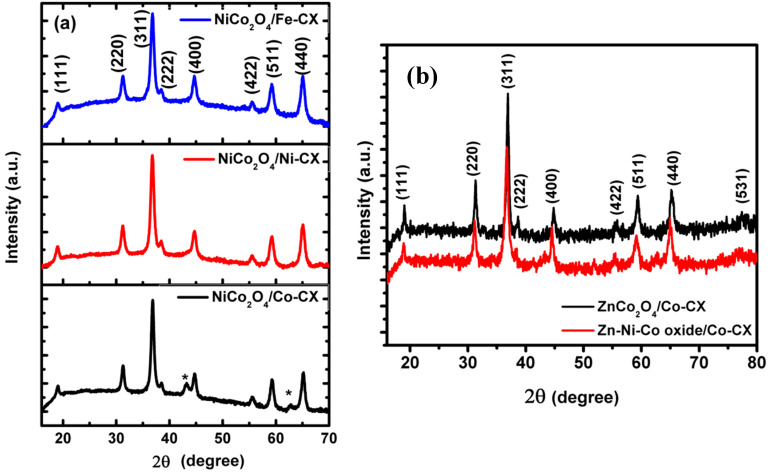
XRD pattern for (**a**) NiCo_2_O_4_/M-CX and (**b**) ZnCo_2_O_4_/Co-CX and Zn-Ni-Co oxide/Co-CX.

The samples’ crystallinity was investigated with powder X-ray diffraction (XRD) and their corresponding patterns are given in Figure 2. The XRD spectra for nickel cobaltite NiCo_2_O_4_ functionalized carbon xerogel are similar to those indexed for the spinel structure of NiCo_2_O_4_, as shown in Figure 2a [37]. There is an excess diffraction peak for NiCo_2_O_4_/Co–CX at 2θ of 43°, which is corresponding to the (200) plane. The XRD spectra for ZnCo_2_O_4_/Co–CX and Zn–Ni–Co oxide/Co–CX, as shown in Figure 2b, are identical with diffraction peaks centered at 2θ of 18.7°, 30.9°, 36.5°, 44.5°, 59.0°, and 64.7°. The diffraction peaks for Zn–Ni–Co oxide/Co–CX are slightly shifted, and there is a higher intensity of the (400) plane at 2θ of 44.5°, indicating the formation of zinc/nickel co-doped Co_3_O_4_ on the carbon xerogel [14].

The mean particle size for the prepared sample is calculated by applying the Scherrer equation to the XRD patterns, as shown in Table 2. Nickel cobaltite-functionalized carbon xerogels have a similar particle size ranging between 21.1 and 25.5 nm. However, there is a remarkable increase in the particle size to 39.2 nm and 38.8 nm when carbon xerogel was doped with zinc cobaltite and Zn–Ni–Co oxide, respectively.

**Figure 3:**
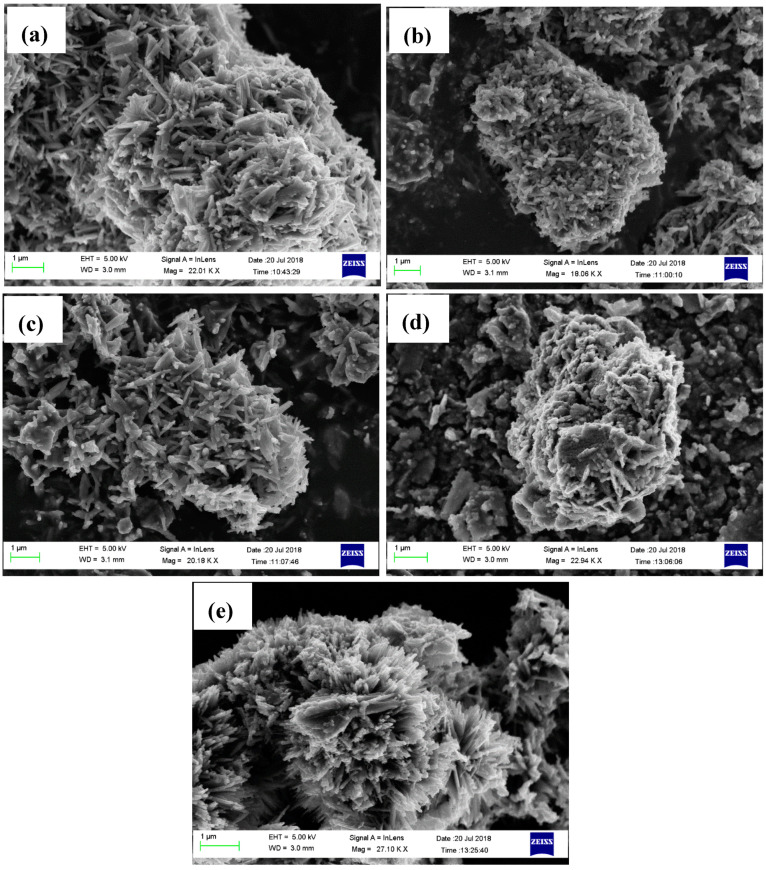
SEM images for (**a**) NiCo_2_O_4_/Co–CX, (**b**) NiCo_2_O_4_/Ni–CX, (**c**) NiCo_2_O_4_/Fe–CX, (**d**) ZnCo_2_O_4_/Co–CX, and (**e**) Zn–Ni–Co/Co–CX.

**Figure 4:**
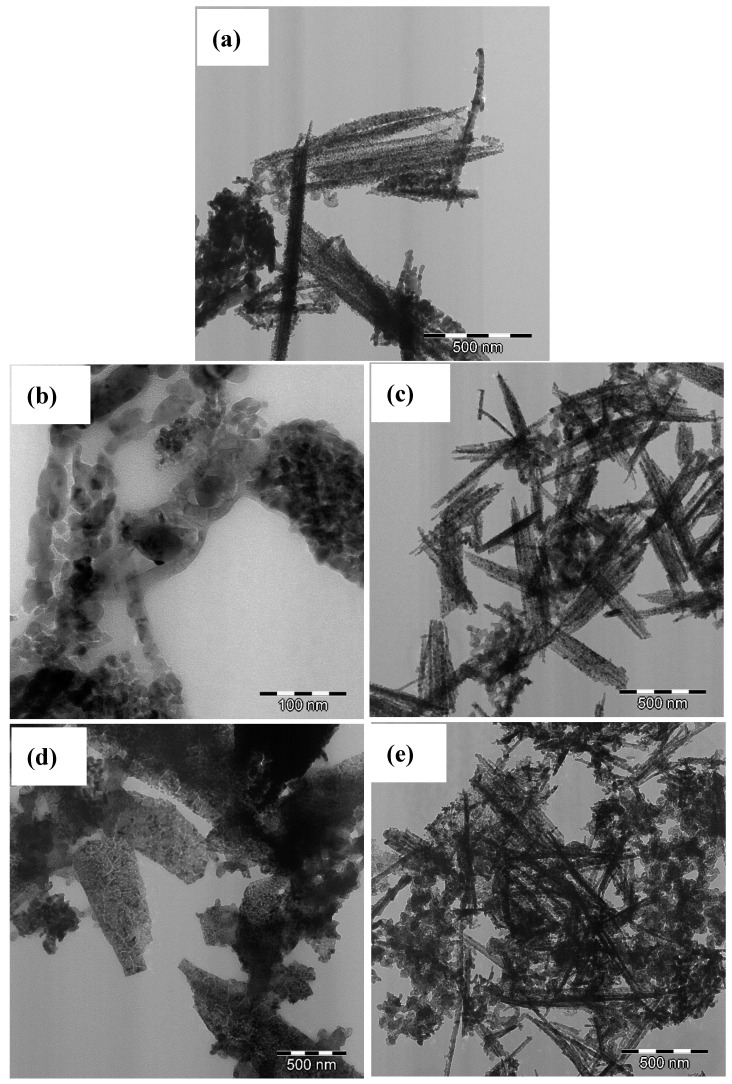
TEM images for (**a**) NiCo_2_O_4_/Co–CX, (**b**) NiCo_2_O_4_/Ni–CX, (**c**) NiCo_2_O_4_/Fe–CX, (**d**) ZnCo_2_O_4_/Co–CX, and (**e**) Zn–Ni–Co/Co–CX.

The samples’ morphology was characterized using scanning electron microscopy (SEM), as shown in Figure 3. SEM images for all of the as-obtained samples clearly show that the samples are composed of 3D nanobundles with different lengths [37,38]. The formation of metal oxides nanobundles can be explained as follows: firstly, the metal cations were adsorbed onto the carbon xerogel surface with the aid of urea as a precipitator agent. During the hydrothermal process, the metal hydroxides were formed in a one-step process and converted into spinel metal oxides nanobundles during the calcination treatment [37]. The nanobundle structure of the prepared samples is also confirmed with TEM analysis. Figure 4 shows the anchoring of nanobundles on the surface of carbon xerogels (Figure 4a–e). In addition, the nanobundles have different widths, ZnCo_2_O_4_/Co–CX being the widest (Figure 4d), whereas the Zn–Ni–Co/Co–CX composite looks completely the opposite (Figure 4e). This difference could be due to the absence of Ni in the first case.

In addition, Figure 4b shows that the NiCo_2_O_4_ nanobundles are composed of many small nanoparticles of 10–20 nm in diameter, indicating that the prepared nanobundles are porous nanobundles instead of single-crystal nanobundles.

Table 3 shows the XPS spectra binding energies (B.E) of carbon, oxygen, and nitrogen atoms, in which the lowest binding energy of oxygen (O1s) at B.E of 529.3 ± 0.3 eV is compatible to oxygen bonded to transition metal cations with oxidation states of +2 and +3, the peaks full width at half maximum (FWHM) is also presented [32].

**Figure 5:**
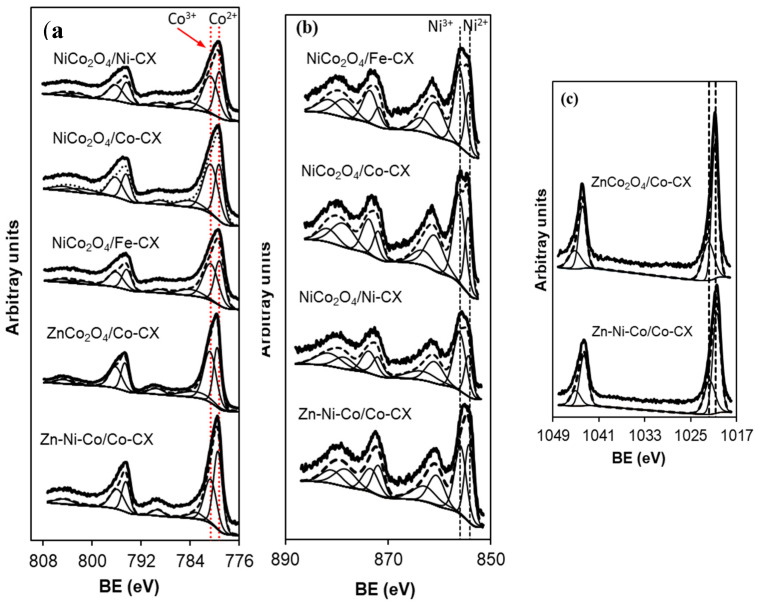
Deconvolution of XP spectra for (**a**) Co2p, (**b**) Ni2p, and (**c**) Zn2p regions.

On the other hand, Table 4 shows the XPS spectra binding energies (B.E) of the metal atoms. In analyzing Co_2p_, as shown in Figure 5a, different Co2p_3/2_ peaks are obtained and assigned as following. In the case of NiCo_2_O_4_-based samples, the signals at 779.2 ± 0.1 correspond to Co^2+^ situated in octahedral holes, whereas the signals at 780.6 ± 0.2 correspond to Co^3+^ situated in tetrahedral holes [39,40]. On the other hand, the XPS spectra of the Ni_2p_ region, as shown in Figure 5b, show signals centered at 854.2 ± 0.1 and 871.7 ± 0.2 eV for the 2p_3/2_ and 2p_1/2_ components respectively, which correspond to Ni^2+^ species, as well as signals at 855.9 ± 0.3 and 873.5 ± 0.2 eV corresponding to Ni^3+^ cations [40]. Iron was not detected in any sample by XPS, and Figure 5c shows the Zn2p spectra. The presence of zinc cobaltite, ZnCo_2_O_4_, is confirmed by a peak centered at 1020.7 eV [41], while the peak at 1021.5–1021.8 eV is assigned to ZnO [42].

Finally, Table 5 collects the superficial chemical composition of the samples. The nitrogen content of the external surface is extremely low in all the samples. Besides, it should be mentioned that Zn-containing samples have the highest total metal concentration in the external surface area, and part of the oxygen content can be due to the calcination treatment during the preparation of the samples. Besides, the ZnCo_2_O_4_/Co–CX composite has a very large Co concentration in the external surface area, which indicates a non-homogeneous metal distribution. This would be able to affect its electrocatalytic performance negatively.

### 3.2. Oxygen Reduction Reaction Activity

**Figure 6:**
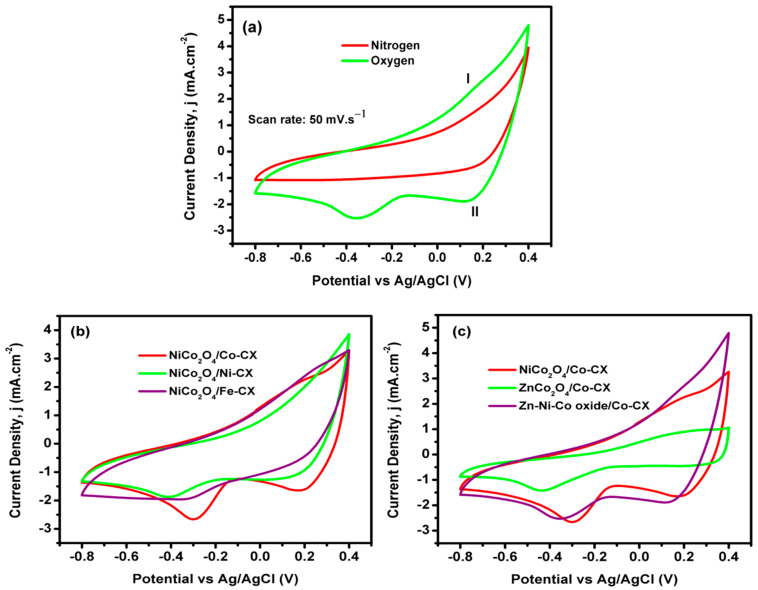
Cyclic voltammograms (CV) of (**a**) Zn–Ni–Co/Co–CX in both nitrogen and oxygen saturated electrolyte, (**b**) NiCo_2_O_4_/M–CX and (**c**) NiCo_2_O_4_/Co–CX, ZnCo_2_O_4_/Co–CX, Zn–Ni–Co/Co–CX in oxygen saturated electrolyte.

The samples’ performance in the presence and absence of oxygen was studied using cyclic voltammetry in oxygen and nitrogen-saturated electrolyte (0.1 M KOH), respectively. Figure 6a shows the behavior of the Zn–Ni–Co/Co–CX sample in saturated oxygen and nitrogen electrolyte at a scan rate of 50 mV∙s^−1^. The figure shows the presence of an oxygen reduction peak, indicating the activity of the used material for ORR. In addition, there are a couple of redox peaks (I/II) at the positive potential range; these peaks come from the reversible transition between Co_3_O_4_ and CoOOH [43]. This reaction can be formulated as Co_3_O_4_ + OH^−^ + H_2_O ↔ 3CoOOH + e^−^. This reduction peak was also detected with all the electrocatalysts; i.e., Figure 6b,c show the compared cyclic voltammograms of the five samples. Thus, regarding the dopant metal (M = Co, Ni, Fe) in the carbon xerogels, the ORR results obtained with the three doped samples functionalized with NiCo_2_O_4_ can be compared in Figure 6b. The results show that the electrochemical performance toward ORR is higher for cobalt-doped carbon xerogels (Co-CX) followed by samples doped with iron (Fe) and nickel (Ni), which is in good agreement with previously published work [31,44]. In general, the incorporation of transition metal into the carbon structure promotes the catalytic activity for ORR due to the generation of more active sites [45]. Related work attributed the lower activity of nickel-doped carbon electrocatalysts to the formation of a nickel oxide NiO on the carbon surface, which has an inhibitory effect on the catalytic current and a poisoning effect on the ORR activity [46].

Another work explains the higher activity of cobalt-based carbon for ORR to the formation of cobalt–nitrogen (Co–N) complexes during the pyrolysis under nitrogen in the preparation of carbon; these Co–N complex moieties have a strong activity for ORR [47]. In any case, we have to consider that the NiCo_2_O_4_/Fe-CX sample has the lowest BET surface area and pore volume (Table 4), as well as a much lower superficial metal content (Table 1) than the other two compared samples; therefore, the electrocatalytic activity promoted by Ni cannot be undervalued at first glance.

**Figure 7:**
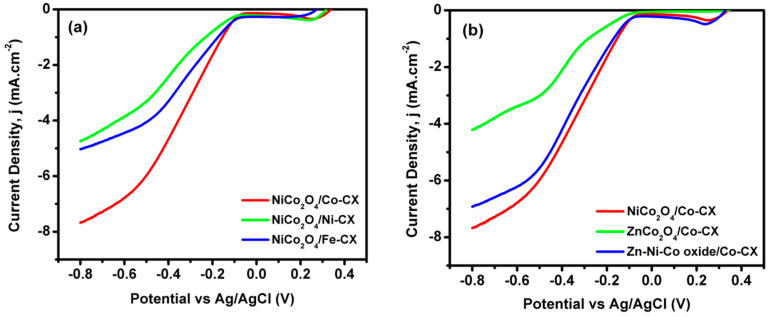
Linear sweep voltammograms (LSV) for (**a**) NiCo_2_O_4_/M-CX and (**b**) NiCo_2_O_4_/Co-CX, ZnCo_2_O_4_/Co-CX, and Zn-Ni-Co oxide/Co-CX at 4000 rpm.

**Figure 8:**
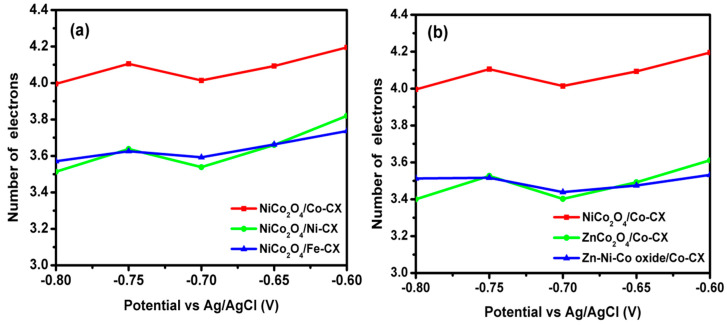
Variation of number of electron transferred with E vs. Ag/AgCl for (**a**) NiCo_2_O_4_/M-CX and (**b**) NiCo_2_O_4_/Co-CX, ZnCo_2_O_4_/Co-CX, and Zn-Ni-Co oxide/Co-CX.

**Figure 9:**
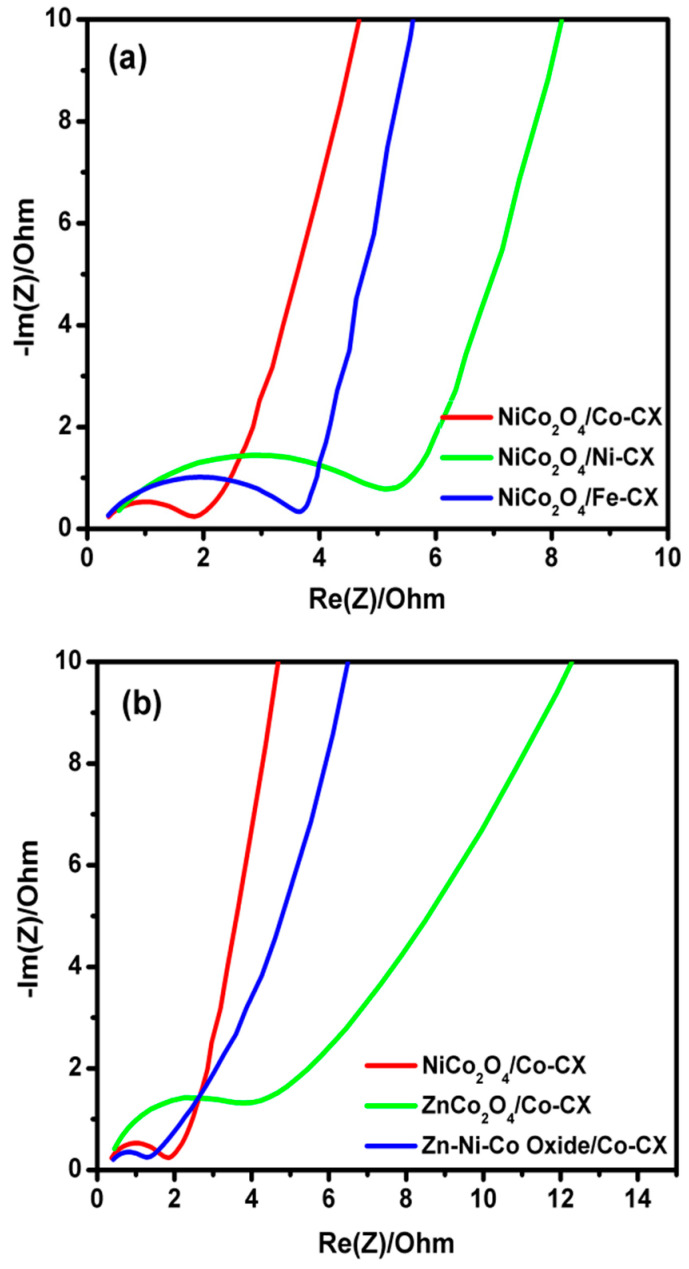
Nyquist plots obtained from electrochemical impedance spectroscopy (EIS) for (**a**) NiCo_2_O_4_/M-CX and (**b**) NiCo_2_O_4_/Co-CX, ZnCo_2_O_4_/Co-CX, and Zn-Ni-Co oxide/Co-CX.

In this way, when the ORR results among all samples containing Co–CX xerogel are compared, the two samples that contain Ni show the best electrocatalytic behavior (Figure 7b). However, this discussion, apart from comparing the quantitative electrochemical parameters, needs to take into account the role of Zn as well. For a good comparison, data obtained from linear sweep voltammetry (LSV) as well as Nyquist plots have been collected in Table 6. Thus, the estimated number of electrons transferred at a given potential obtained from the Koutecky–Levich equation are all good enough to work well as electrocatalysts for ORR, and they also keep these values at different potentials (Figure 8). The number of electrons transferred can proportionally vary a little bit depending on the type of electrode used, and if a low formation of peroxides occurs, which in this work has not been analyzed. Besides, the number of electrons transferred has a direct relation and depends on the electrocatalyst resistance. The electrochemical impedance spectroscopy (EIS) is an important technique that determines the behavior of an electrode in certain application and its resistance. Figure 9 shows the Nyquist plots for the prepared electrodes. As it can be seen, a semicircle is observed at the high-frequency region due to charge transfer resistance on the electrode/electrolyte interface. Meanwhile, at low frequency, a purely capacitive behavior is obtained, and the combination between both the resistive and capacitive behavior is known as the Warburg region, which has a relation to the diffusive resistance [37,48]. The equivalent series resistance (ESR) of the electrode can be determined by the intersection of the semicircle with the real axis.

In the Nyquist plots for all composites (Figure 9), the diameter of the semicircle for Zn–Ni–Co/Co–CX is the lowest, indicating the lowest charge-transfer resistance (R_ct_) between the electrode and electrolyte interface. Moreover, the lowest equivalent series resistance (ESR) is obtained for Zn–Ni–Co/Co–CX, indicating the better conductivity of this electrocatalyst. On the other hand, we should remark on the low values of E_onset_ among all our electrocatalysts, showing again the Zn–Ni–Co/Co–CX composite as one of the smallest. Moreover, this composite exhibits the best electrocatalytic behavior in ORR in spite of its much larger mean metal particle size and lower surface area and pore volumes.

The available bibliography about a possible effect of the metal particle sizes on the ORR activity is not clear. Some works with Pt supported on carbon materials found a relation between the particle size and the specific activity toward ORR. However, while some of them mention that for oxygen reduction, a loss of catalytic activity with the decreased Pt particle size occurs due to the stronger adsorption of oxygenated species [12,49], other authors found an optimal Pt particle size [50]. Nevertheless, with Fe, Co, or Ni-based catalysts, the previous mentioned tendencies seem to be different, where the small-sized cobalt nanoparticles and dispersion in carbon provide fast electron transport between the carbon matrix and the cobalt nanoparticles, leading to efficient electrical conductivity [13,51]. However, in that work, the samples did not contain Zn. Therefore, it could very well be that the extremely large main crystallite sizes of samples containing Zn can be more of a handicap than an advantage. However, the Zn–Ni oxide phases combination seems to improve the electrocatalytic behavior, especially that related with the conductivity of the electrode. On the other hand, the ZnCo_2_O_4_/Co–CX composite, due to its very large Co concentration in the external surface area and large mean metal particle size, partially loses the synergism with the doped xerogel support, showing the worst electrocatalytic performance. This means that the sizes of these types of metal phases should be better controlled and optimized for future applications.

Finally, reviewing the available literature, we have found that the higher electrochemical activity for ORR is obtained by comparing our results with already published ones at similar conditions. For example, Zhang et al. [52] report a composite NiCo_2_O_4_/N–rGO with onset potential, E_onset_, of about −0.08 V, while it is just −0.06 V versus the Ag/AgCl reference electrode for two of our best composites. Moreover, when comparing our ternary metal oxide electrocatalyst Zn–Ni–Co oxide/Co–CX with a published nanoalloy of Pt_25_Ni_16_Co_59_/C [53], a higher ORR current density is obtained of about −8.0 mA∙cm^−2^ compared with −6.0 mA∙cm^−2^ for Pt_25_Ni_16_Co_59_/C at similar conditions.

## 4. Conclusions

Different combinations of Zn–Ni–Co oxide phases forming 3D nanobundles were homogeneously developed on doped carbon xerogel surfaces by the hydrothermal method. The type of carbon xerogel dopant plays an important role in ORR activity, in which cobalt-doped carbon xerogels have higher electrochemical ORR activity than Fe or Ni. All composites show excellent activity to oxygen reduction reaction, and the low equivalent series resistance that the Zn–Ni–Co/Co–CX composite has is especially remarkable, indicating a high electronic conductivity. All composites show very low onset potentials, E_onset_, ranging between −0.06 and −0.09 V. Overall, the electrocatalytic behavior of this composite is very promising in spite of its lower surface area and pore volumes as well as its large mean metal particle size, because all these parameters can be optimized and improved. Therefore, the role of Zn in this type of metal oxides nanobundles-based ORR catalyst is not only positive, but its effect could be enhanced by the presence of Ni.

## Figures and Tables

**Table 1:** Textural characteristics of the samples.

Sample	S_BET_	W_0_ (N_2_)	L_0_ (N_2_)	V_0.95_ (N_2_)	V_meso_ (N_2_)	S_DFT_	V_DFT_	L_0_ (DFT)
	m^2^/g	cm^3^/g	nm	cm^3^/g	cm^3^/g	m^2^/g	cm^3^/g	nm
NiCo_2_O_4_/Co–CX	64	0.03	2.98	0.30	0.27	128	0.32	2.84
NiCo_2_O_4_/Ni–CX	57	0.02	2.20	0.27	0.24	103	0.31	2.60
NiCo_2_O_4_/Fe–CX	74	0.03	3.23	0.44	0.41	201	0.38	2.84
ZnCo_2_O_4_/Co–CX	57	0.02	2.35	0.27	0.25	122	0.25	2.60
Zn–Ni–Co/Co–CX	44	0.02	1.73	0.21	0.19	79	0.18	2.60

**Table 2:** Mean particle size obtained from applying the Scherrer equation to XRD patterns.

Sample	*d*_XRD_ (nm)
NiCo_2_O_4_/Co–CX	25.5
NiCo_2_O_4_/Ni–CX	24.1
NiCo_2_O_4_/Fe–CX	21.1
ZnCo_2_O_4_/Co–CX	39.2
Zn–Ni–Co oxide/Co–CX	38.8

**Table 3:** Binding energies of C1s, O1s, and N1s regions.

Sample	C1s	FWHM	Peak	O1s	Peak	N1s
	eV	eV	%	eV	%	eV
NiCo_2_O_4_/Co–CX	284.6	1.4	70.9	529.3	36.7	399.3
	285.7	–	8.9	530.7	24.7	400.7
	286.3	–	9.7	531.8	23.3	–
	288.5	–	10.4	533.2	15.3	–
NiCo_2_O_4_/Ni–CX	284.6	1.4	67.5	529.1	34.9	398.9
	285.6	–	11.0	530.7	27.8	400.4
	286.4	–	10.4	531.8	22.4	–
	288.5	–	11.1	533.2	14.9	–
NiCo_2_O_4_/Fe–CX	284.6	1.4	70	529.2	28	399.3
	285.8	–	11	530.6	22	400.7
	286.6	–	8	531.8	28	–
	288.6	–	11	533.2	22	–
ZnCo_2_O_4_/Co–CX	284.6	1.1	65	529.6	55	399.3
	285.4	–	16	530.6	23	400.7
	286.4	–	13	531.7	16	–
	288.5	–	7	533.2	6	–
Zn–Ni–Co/Co–CX	284.5	1.2	61	529.2	46	399.3
	285.4	–	18	530.7	25	400.3
	286.4	–	12	531.8	18	–
	288.6	–	9	533.2	10	–

**Table 4:** Binding energies (eV) of Co2p, Ni2p, and Zn2p regions.

Sample	Co2p_3/2_	Peak (%)	Ni2p_3/2_	Peak (%)	Zn2p_3/2_	Peak (%)
NiCo_2_O_4_/Co–CX	779.2	43.0	854.3	34.4	–	–
	780.6	57.0	856.1	65.6	–	–
	783.9	–	860.9	–	–	–
	788.8	–	863.2	–	–	–
	794.4	–	871.9	–	–	–
	796.2	–	873.7	–	–	–
	801.6	–	878.7	–	–	–
	804.5	–	881.7	–	–	–
NiCo_2_O_4_/Ni–CX	779.2	43.7	854.2	32.3	–	–
	780.6	56.3	856.0	67.7	–	–
	784.1	–	860.8	–	–	–
	788.8	–	863.5	–	–	–
	794.4	–	871.7	–	–	–
	796.2	–	873.5	–	–	–
	802.4	–	878.5	–	–	–
	804.8	–	881.5	–	–	–
NiCo_2_O_4_/Fe–CX	779.2	43.9	854.3	35.5	–	–
	780.6	56.1	856.0	64.5	–	–
	783.9	–	860.9	–	–	–
	788.7	–	863.4	–	–	–
	794.4	–	872.0	–	–	–
	796.1	–	873.7	–	–	–
	801.8	–	878.5	–	–	–
	804.7	–	881.5	–	–	–
ZnCo_2_O_4_/Co–CX	779.5	41.9	–	–	1020.7	67.0
	780.7	58.1	–	–	1021.9	33.0
	783.0	–	–	–	–	–
	789.5	–	–	–	–	–
	794.7	–	–	–	–	–
	796.2	–	–	–	–	–
	802.4	–	–	–	–	–
	804.7	–	–	–	–	–
Zn–Ni–Co/Co–CX	779.4	53.0	854.2	45.3	1020.5	67.7
	780.7	47.0	855.8	54.7	1021.8	32.3
	783.0	–	860.5	–	–	–
	789.3	–	862.8	–	–	–
	794.5	–	871.9	–	–	–
	795.9	–	873.4	–	–	–
	801.2	–	878.3	–	–	–
	804.6	–	880.9	–	–	–

**Table 5:** Surface chemical composition in wt % obtained by XPS.

Sample	C	O	N	Co	Ni	Zn
NiCo_2_O_4_/Co–CX	37.3	28.8	0.2	21.6	12.1	0.0
NiCo_2_O_4_/Ni–CX	40.3	29.6	0.2	18.7	11.2	0.0
NiCo_2_O_4_/Fe–CX	46.8	27.4	0.1	17.5	8.2	0.0
ZnCo_2_O_4_/Co–CX	20.0	30.7	0.2	41.7	0.0	7.4
Zn–Ni–Co/Co–CX	28.5	28.5	0.2	21.5	11.6	9.7

**Table 6:** Parameters obtained from LSV @ 4000 rpm (values of n refer to K-L fitting for data at −0.8 V) and equivalent series resistance (ESR) calculated from the Nyquist plot.

Sample	E_onset_	n	ESR
	V		Ω
NiCo_2_O_4_/Co–CX	−0.06	4.0	2.74
NiCo_2_O_4_/Ni–CX	−0.07	3.5	6.18
NiCo_2_O_4_/Fe–CX	−0.07	3.6	3.90
ZnCo_2_O_4_/Co–CX	−0.09	3.4	4.63
Zn–Ni–Co/Co–CX	−0.06	3.5	1.80
